# Criss-cross

**DOI:** 10.1007/s12471-018-1101-9

**Published:** 2018-03-08

**Authors:** R. van der Nagel, V. F. van Dijk

**Affiliations:** 0000 0004 0622 1269 grid.415960.f St. Antonius Ziekenhuis, Nieuwegein, The Netherlands

A 50-year-old male patient of Eastern European origin was admitted to our clinic for aortic valve replacement surgery because of severe stenosis of a bicuspid aortic valve. Recently he had been diagnosed with a third degree atrioventricular block, for which a dual-chamber pacemaker (Medtronic Advisa DR MRI A3DR01) was implanted at our clinic.

After aortic valve replacement surgery, the device was routinely interrogated. In the Quick Look screen, multiple episodes of atrial tachycardia/atrial fibrillation were annotated. All episodes resembled the recording/dot blot in Fig. 1.

**Fig. 1:**
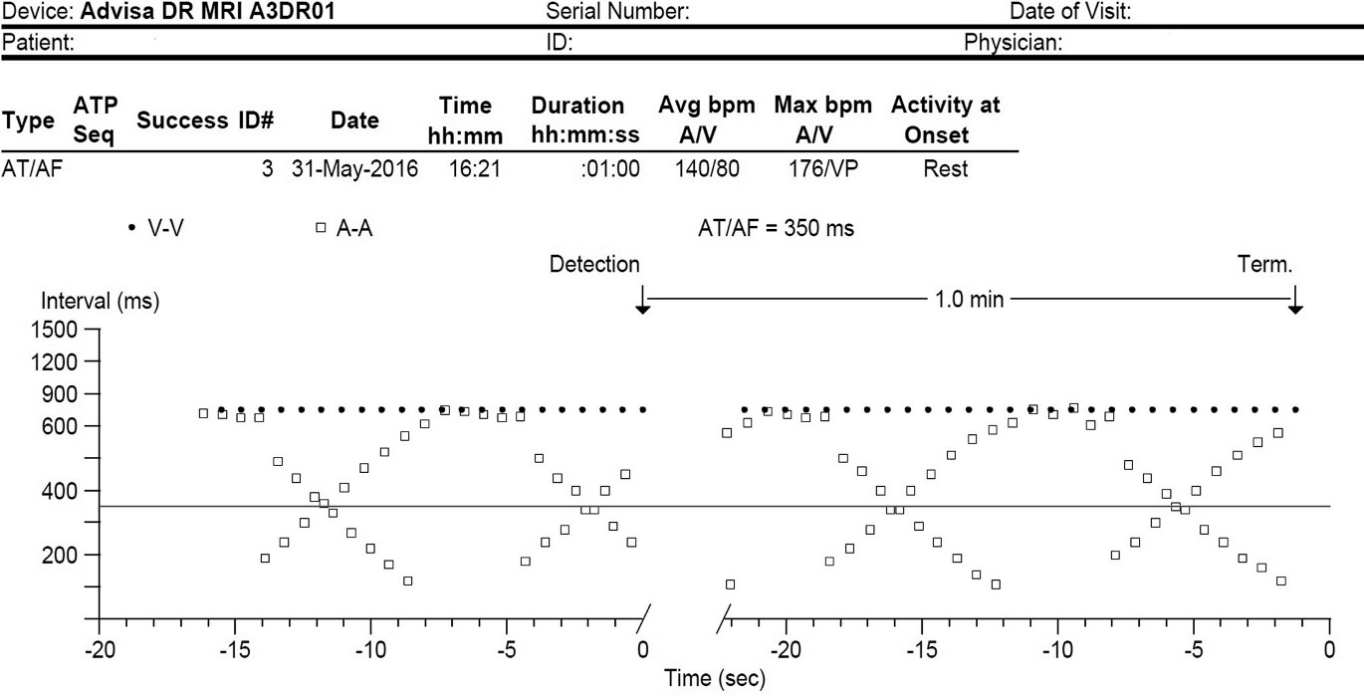
Dot blot of a typical episode

What is the explanation of the peculiar pattern?

## Answer

You will find the answer elsewhere in this issue.

